# The mental health crisis: Only top down regulation will cure medicine’s folly

**DOI:** 10.1371/journal.pmen.0000439

**Published:** 2025-09-22

**Authors:** Robert Charles Smith

**Affiliations:** 1 University Distinguished Professor Emeritus of Medicine and Psychiatry, Department of Medicine, Michigan State University, Battle Creek, Michigan, United States of America; PLOS: Public Library of Science, UNITED KINGDOM OF GREAT BRITAIN AND NORTHERN IRELAND

Americans face a mental health crisis. Only 25 percent of individuals receive any treatment at all [1,2], compared to 60–80 percent of patients with physical disorders [2,3]. And, of those who do receive care, primary care physicians provide 75 percent of it [4–6]; all mental health professionals combined conduct the remaining 25 percent of care, psychiatry only 12 percent [7]. Yet research consistently demonstrates that primary care doctors miss most mental health problems and, when they provide care, too often prescribe the wrong medication, with little or no follow-up, and omit nondrug options such as addressing lifestyle issues [5,6,8]. This has huge policy implications: mental illnesses are the nation’s most common health condition, afflicting over 90 million people [9].

But it’s not the doctors’ fault. US medical schools and residencies devote no more than 2 percent of total training time to mental health and other relevant psychosocial issues such as empathy, doctor-patient communication, and lifestyle issues [10]. The omission is not an oversight but a structural choice by medicine’s governing bodies, the Association of American Medical Colleges (AAMC) and the Accreditation Council for Graduate Medical Education (ACGME). Predictably, graduates lack the skills and attitudes to address the very conditions most of their patients face.

But the damage doesn’t end with mental illnesses. Excluding training in psychological and social medicine jeopardizes care for physical diseases. For example, thirty million Americans with a chronic physical disorder have a comorbid mental health problem that doctors must treat if the physical problem is to improve [11]. And omission of psychosocial issues from teaching leaves learners shorthanded when addressing the lifestyle issues (alcohol, other substances, and tobacco use; obesity; lack of exercise; stress) that cause 80 percent of all cardiovascular disease and diabetes and 40 percent of all cancer [12], thus precluding both treatment and prevention. Because chronic diseases account for 75 percent of all US health care spending [12], we could save half or more of the annual $5 trillion healthcare expenditures with effective prevention. Now, of course, as part of its 2 percent of training time, medical education does address key issues such as suicide, chronic pain, depression, anxiety, substance abuse, and lifestyle issues. But it’s only a few lectures here and there. There’s no supervised training with real patients experiencing these problems.

We do have an explanation for this bizarre, counterproductive behavior that jeopardizes mental and physical health, wasting trillions of dollars each year along the way. This understanding points not only to specific policy changes that must occur to better serve the public—but also to scientific changes.

In the 16^th^ and 17^th^ centuries Scientific Revolution, the church forced medicine to dissociate the mind from the body in teaching. As part of the emergence from its dark ages, medical science sought to better understand human anatomy, realizing its reliance on dissections of pigs, goats, and cattle likely provided an inaccurate representation of humans. But the all-powerful church had prohibited human dissections for over a millennium. Then, in 1537 Pope Clement finally allowed dissections, but he forbade examination of the head, which the church viewed as the source of the mind, spirit, and soul. Medicine could then dissect the body from the neck down. That launched what we now call the “mind-body split” [10].

A century later, the even more powerful influence of philosophers Descartes, Locke, Hobbes, and others firmly established that mind issues were off base for medicine. The mind-body duality emerged in clinical medicine in Europe in the early 1800s with the discovery in pathology that the abnormal appearing organs and tissues found at autopsy represented diseases and caused physical symptoms. We take clinical pathological correlation for granted today, but it was revolutionary at the time, supplanting the greater than two millennia-old four humors (black bile, yellow bile, blood, phlegm) theory of disease [10].

But treatment of physical diseases did not improve until the last century when life survival dramatically doubled from 40 years in 1900 to nearly 80 years by 2000. The discovery of penicillin, sulfa, ether, and chloroform was followed by revolutionary developments in care, for example, vaccines, new drugs (statins, beta-blockers, GLP-1 agonists), MRI, organ transplants, the genome, and AI. The mind-body split theory succeeded famously [10].

But success bred failure. With people living longer, acute physical disorders were replaced by mental health problems and chronic physical diseases—both requiring integration of the very psychosocial issues medicine shuns. Despite warnings from within the profession dating to the 1970s, medicine perpetuated an isolated physical disease focus via medical education and risked becoming a nonscientific discipline [10].

**Fig 1:**
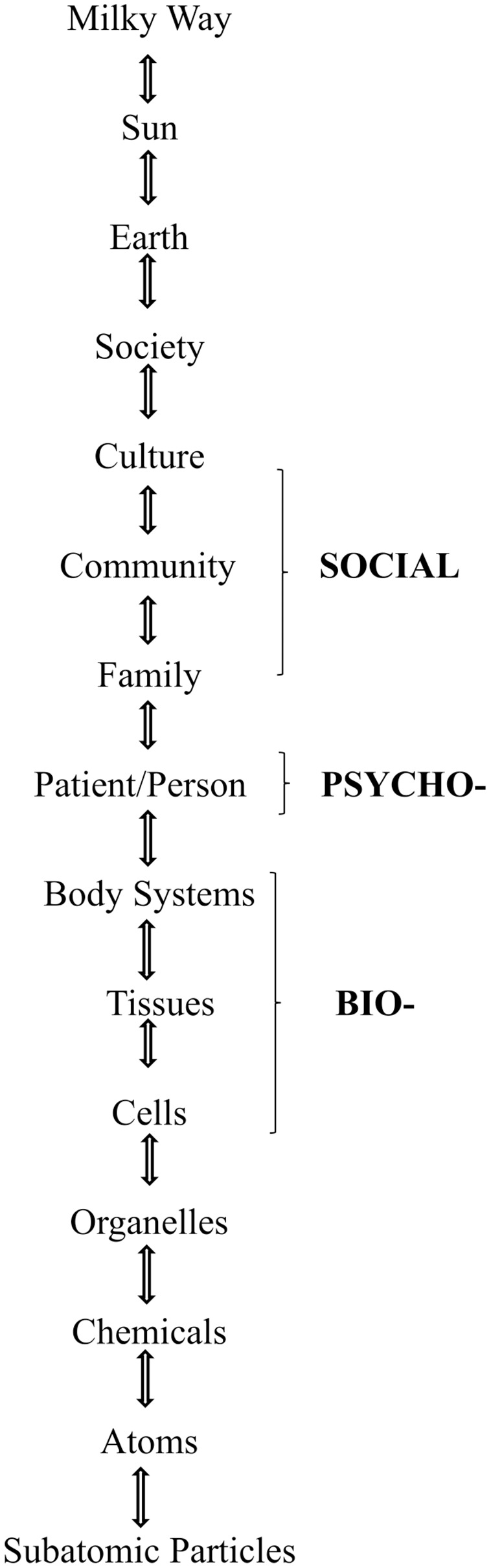
The biopsychosocial model within the systems hierarchy.

Along with medicine, all sciences began with a reductionistic view on only one to two of many parts of their discipline. By the early 1900s, however, all sciences, starting with physics, changed to a new theory: a holistic systems view that includes all parts of its discipline. Only medicine continues a reductionistic focus on physical disease while excluding the very psychological and social features that make the patient human. Importantly, the other sciences did not give up their earlier reductionistic advances. For example, physics complemented Newtonian mechanics with relativity and quantum mechanics. Nevertheless, medicine has repeatedly ignored George Engel’s systems-based model for medicine published in 1977—the biopsychosocial model (Fig 1) [13]. It continues to address just the bio part (disease) of the systems hierarchy [10].

The policy requirements are clear. Medicine must change to a systems-based approach in its teaching, clinical care, and research. But history shows that internal reform is unlikely. Just as our political representatives created the Federal Reserve to protect the public from banking excesses, we need top-down regulation to realign medicine with the realities of 21^st^-century health. Specifically, a federal governing board would work with medical education to ensure that all graduates are as competent with mental disorders as with physical disorders. Its scope, though, would extend beyond education to ensure that systems principles are applied in all medical care, research, and prevention—that medicine implements a new paradigm.
